# Genetic testing in motor neurone disease

**DOI:** 10.1136/practneurol-2021-002989

**Published:** 2022-01-13

**Authors:** Thanuja Dharmadasa, Jakub Scaber, Evan Edmond, Rachael Marsden, Alexander Thompson, Kevin Talbot, Martin R Turner

**Affiliations:** 1 Nuffield Department of Clinical Neurosciences, Oxford University, Oxford, UK; 2 Oxford University Hospitals NHS Foundation Trust, Oxford, UK

**Keywords:** ALS, genetics, motor neurone disease, neurogenetics, clinical neurology

## Abstract

A minority (10%–15%) of cases of amyotrophic lateral sclerosis (ALS), the most common form of motor neurone disease (MND), are currently attributable to pathological variants in a single identifiable gene. With the emergence of new therapies targeting specific genetic subtypes of ALS, there is an increasing role for routine genetic testing for all those with a definite diagnosis. However, potential harm to both affected individuals and particularly to asymptomatic relatives can arise from the indiscriminate use of genetic screening, not least because of uncertainties around incomplete penetrance and variants of unknown significance. The most common hereditary cause of ALS, an intronic hexanucleotide repeat expansion in *C9ORF72,* may be associated with frontotemporal dementia independently within the same pedigree. The boundary of what constitutes a possible family history of MND has therefore extended to include dementia and associated psychiatric presentations. Notwithstanding the important role of clinical genetics specialists, all neurologists need a basic understanding of the current place of genetic testing in MND, which holds lessons for other neurological disorders.

## Introduction

Amyotrophic lateral sclerosis (ALS, the most common form of motor neurone disease, MND) is still a significantly life-shortening disorder for most cases, despite the benefits of specialised multidisciplinary care.^1 2^ The diagnosis of MND is fundamentally clinical, supported by confirmatory electrophysiological findings and negative neuroimaging, and with generally no requirement for extended ancillary investigations.^3^

Since the familial linkage of the superoxide dismutase-1 (*SOD1*) gene to ALS in 1993, variants in many more genes have been implicated in cases of ALS, mostly in an autosomal dominant pattern of inheritance. Substantial progress in sequencing methodology has yielded panels for the simultaneous testing of multiple genes, now accessible in most secondary healthcare settings. Whole-genome sequencing is also entering routine clinical use, as exampled in childhood developmental disorders and epilepsy. Novel therapies targeting specific ALS-determining genes are moving from preclinical development through to clinical trial pipelines. While patients increasingly value the availability and utility of genetic testing—seeking to understand more about their disease and any implications for relatives^4^ —an inconsistent approach by clinicians to testing presents a problem.^5^ The subtleties and complexity of the rapidly expanding genetic information now available for a range of neurological conditions, not just for ALS, requires all neurologists to feel able to navigate this in routine practice. We outline some background considerations and suggest a framework for genetic testing after a diagnosis of ALS has been made clinically.

### Genetics of ALS

Approximately 10%–15% of people diagnosed with ALS report a family history of the disorder, typically with dominant inheritance, but most cases appear to arise sporadically. ALS is the common clinical end point of a multistep pathogenesis that may involve varied upstream events.^6 7^ Within this framework, pathogenic variants (a term increasingly preferred to ‘mutation’) in more than 40 genes have been associated with the broad term ALS, including some cases with atypical phenotypes that diverge from the canonical features associated with consensus criteria.^8^ Although a single pathological gene variant (monogenic) is typical, a small proportion of cases show co-occurrence of variants in more than one gene.^9–11^

**Figure 1:**
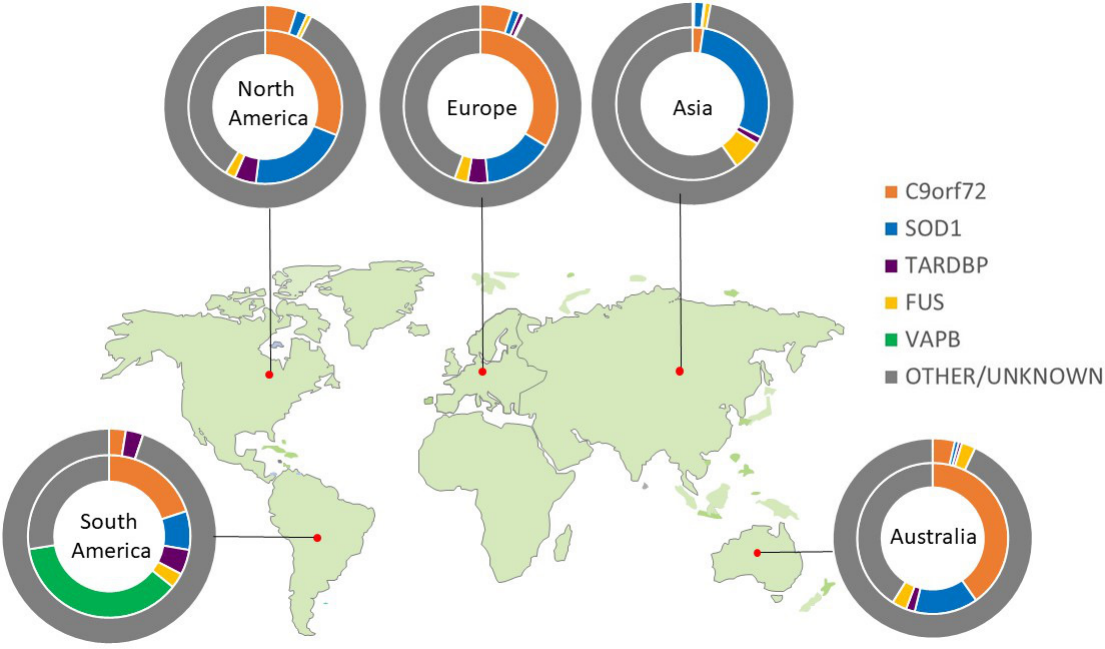
Global genetic architecture of ALS. Charts show proportion of known pathogenic variants within each geographical area. Inner circle calculated from familial cohort; outer circle calculated from apparent sporadic cohort.

Only four genes are commonly linked to familial ALS cases of European ancestry. An intronic hexanucleotide (GGGGCC) repeat expansion in *C9ORF72* accounts for ~40%, variants in *SOD1* for ~20% and *FUS* and *TARDBP* each <5%. Variants in >20 other genes each account for less than 1%. The population frequency of pathological variants differs markedly between geographical regions, spotlighting the importance of ancestry when considering an individual’s genetic risk. For example, the *C9ORF72* hexanucleotide repeat expansion is rare among Asian populations, where *SOD1* variants are the most prevalent,^12^ and *VAPB* is the most common gene in South American populations (figure 1). Despite this genetic heterogeneity, 97% of ALS cases (familial or sporadic) have neuronal and glial cytoplasmic aggregates of phosphorylated TDP-43 at postmortem.^13^ Those linked to *SOD1* and *FUS* are notable exceptions, despite sharing a common clinical syndrome.

Genotype–phenotype correlations are limited to group trends and have low predictive value in individual cases. Patients harbouring a *C9ORF72* hexanucleotide repeat expansion have a slightly younger age of symptom onset (50s vs 60s), a higher rate of bulbar-onset and a more rapid rate of disease progression overall, but none of these features have meaningful pretest predictive value. Crucially however, the *C9ORF72* hexanucleotide repeat expansion is also the most common monogenic cause of frontotemporal dementia (FTD), typically presenting as the behavioural variant. Clinicopathological links between ALS and FTD have long been recognised through shared TDP-43 pathology and a spectrum of similar cognitive and behavioural change.^14^ Pedigrees associated with the *C9ORF72* hexanucleotide repeat expansion may therefore contain cases of relatively ‘pure’ FTD (which may have been labelled as just ‘dementia’ or even erroneously as Alzheimer’s disease), ‘pure’ ALS, or mixed ALS–FTD.^15^ Some of these FTD-involved cases may be associated with psychiatric symptoms, including frank psychosis.^16^ Although these clinical features are likely to be similarly enriched in ‘sporadic’ carriers of the *C9ORF72* hexanucleotide repeat expansion, who probably have obscured familial disease (see later), the literature is inconclusive and hampered by limited studies of small sample sizes. Comparatively, ALS cases linked to variants in *SOD1* tend to result in more lower motor neurone predominant phenotypes and are only rarely associated with cognitive change. Variants in *FUS* have been associated with an often rapidly progressive form of ALS that may present in younger adults, including teenagers.^17^

### Therapeutic developments in genetic forms of ALS

Genetic testing practices differ across countries, influenced by cultural and economic factors, with no formal consensus between MND specialists. While a high proportion (93%) offer testing for those with a positive family history, only one-third report testing apparent sporadic cases.^5 18^ The 2012 European Federation of Neurological Societies guided against testing cases of ‘sporadic ALS with a typical classical ALS phenotype’.^19^ USA practice guidelines in 2009 did not specifically address this issue,^20^ while guidance within the UK National Health Service at the time of writing suggests reserving testing for those with an ALS diagnosis at an age of onset <40 years or a family history of MND or FTD.

The success of gene-targeting therapy in spinal muscular atrophy has raised expectations of the prospect of similar strategies in other neurological disorders, including ALS.^21^ Phase 1 and 2 antisense oligonucleotide trials targeting wild-type *SOD1* have been completed^22^ and several phase 1/2 studies are underway for *C9ORF72* hexanucleotide repeat expansion-associated ALS. Exclusion criteria for these current gene directed trials include poor respiratory function and concurrent illness (infection/immunodeficiency), but this has not yet been of significant influence on testing decisions in the clinic, at least while such trials are in their infancy. Any perceived deterrence this may have on genetic testing decisions for the patient is also likely to be negated by the future hope of potential genetic therapy success. With the realisation that the emergence of motor symptoms represents the late stage of ALS pathology, there is now interest in using a presymptomatic rise in blood neurofilament light chain in *SOD1* variant carriers to trigger initiation of antisense oligonucleotide therapy. Increasing awareness of the potential for genetic therapy is therefore likely to lead to more widespread use of genetic testing.

### What constitutes a family history?

For most ALS-associated genes, potential pathological variants have been identified in both familial and apparent sporadic cases. This is strikingly true of the two commonest ALS genes, *SOD1* and *C9ORF72*, where the proportion of mutation carriers with no family history may be as high as 50% and 85%, respectively.^23^ These typically represent previously unrecognised familial cases due to an incomplete family history rather than de novo mutations, although this can occur, as seen in the case of *SOD1*.^24^ Despite classification of ALS by the terms familial and sporadic thus becoming increasingly redundant, this still forms the framework used by many clinicians to screen for genetic mutations. However, there is no consistent definition on what constitutes a relevant family history in ALS- for example, the presence of ALS or FTD in one or more first-degree relatives or, less certainly, in one or more second-degree relatives.^25 26^ Ascertainment bias and family size also play a direct role in whether an individual case appears to be familial or sporadic,^27 28^ and incomplete penetrance is another important consideration (see later).

**Figure 2:**
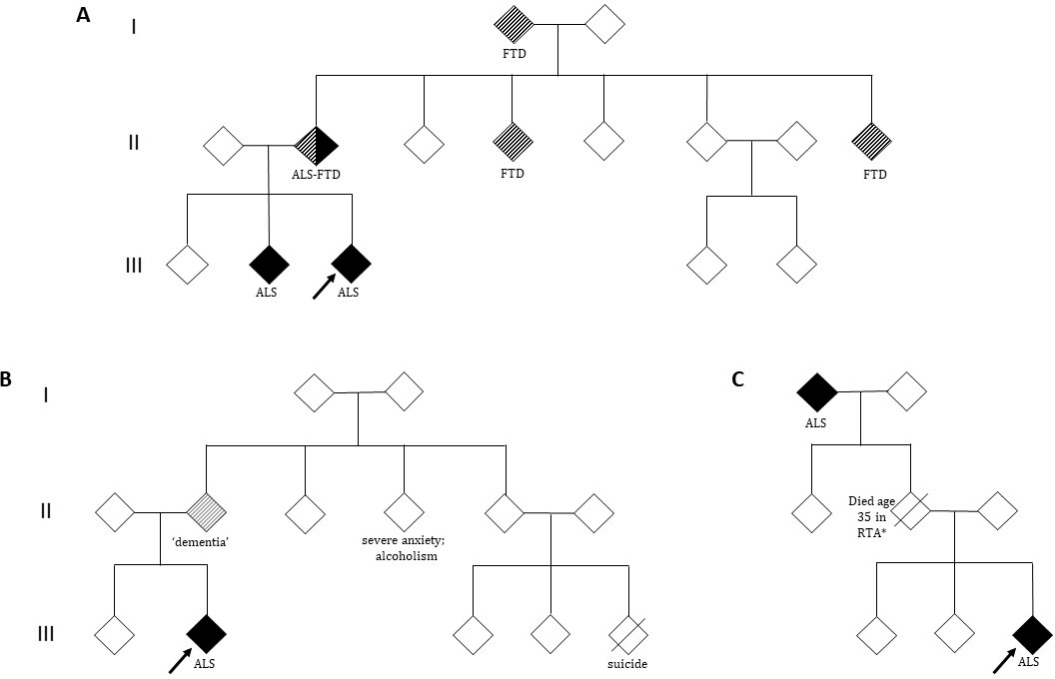
Patient pedigrees. Index patient shown by arrow; white symbols, unaffected individuals; diagonal line, deceased. (A) Pedigree showing an autosomal dominant inheritance of ALS. *C9ORF72* hexanucleotide repeat expansion pedigrees may contain cases of FTD, ALS or mixed ALS–FTD independently within the same lineage. (B) The history of dementia in a parent must be explored further in this family, with relevance of this dependent on how suggestive this case is of FTD(for example, prominent behavioural changes). Other neuropsychiatric conditions, although not predictive in isolation, may also feature within an ALS kindred (particularly in the context of the *C9ORF72* hexanucleotide repeat expansion). (C) The inheritance pattern may be obscured by the early death of a parent (*eg, RTA, road traffic accident) and the disease appears to skip a generation. There are more complex issues around variable penetrance to consider, including non-paternity, and it is also possible for an asymptomatic carrier parent to have a child who dies of ALS before they themselves develop symptoms. ALS, amyotrophic lateral sclerosis; FTD, frontotemporal dementia.

It is critical to map out the pedigree of someone diagnosed with ALS in detail, even if information is incomplete (figure 2). Inheritance patterns may be obscured by the early death of parents from other causes (before the typical age of onset of ALS or FTD), unclear parentage, estrangement or undisclosed family diagnoses. The history must explore disorders that share a common genetic background with ALS. FTD is the most important of these, largely underpinned by the *C9ORF72* hexanucleotide repeat expansion but also by variants in *TBK1*, for example. Differentiating FTD from the more common cases of late-life Alzheimer’s disease is challenging. A strong clue to consider FTD is a younger age of symptom onset (50s or 60s vs 70s or 80s), particularly with prominent behavioural changes rather than memory difficulties. The former may include apathy, disinhibition and sweet food preference.^29^ Neuropsychiatric conditions may also cluster in ALS kindreds, particularly in the context of the *C9ORF72* hexanucleotide repeat expansion, and include bipolar disorder, schizophrenia, drug dependence and autism-spectrum disorders,^30–32^ though these are less predictive in isolation. Population-based studies show that within the 9% carrier rate of *C9ORF72* hexanucleotide repeat expansion from people diagnosed with neurodegenerative disease, more than one-third present with a non-ALS condition.^33^

### Challenges of genetic sequencing interpretation

Growth in the quantity and complexity of available genetic information has introduced new challenges for clinical interpretation, which will expand further with more common use of whole-genome sequencing.

#### Incomplete penetrance

**Figure 3:**
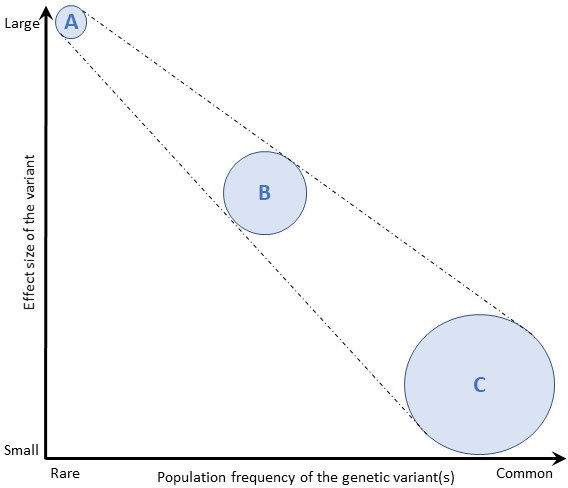
Effect size of genetic variants. The population frequency of genetic variants is depicted against their effect size. (A) Rare variants are more likely to impart larger effect sizes then (C) common variants, which typically impart small (or clinically negligible) effects. Rare variants (A) with very large effect sizes cause single-gene diseases, multiple uncommon variants with moderate effect sizes (B) cause oligogenic diseases and a very large number of common variants (C) are responsible for diseases with more complex genetic contributions. Adapted from Marian *et al*, 68(25);2016.

As pathogenic variants associated with ALS commonly display autosomal dominant inheritance, this translates to a 50% carrier risk for a first-degree relative (i.e., biological sibling or child) of the patient. The risk of developing the clinically manifest disease, however, depends on the penetrance rate (the probability that a specific phenotype will be expressed by the individual carrying the risk genotype) and the effect of the genetic variant on phenotype (effect size). In general, a rare variant (i.e., a variant with a frequency of <1% in the population) is more likely to be pathogenic than a common variant, and typically displays a large effect size and high penetrance. A single such mutation is usually sufficient to cause disease. By contrast, most variants identified through genome-wide association studies are common variants, which appear to have small (or negligible) clinical effects on their own, usually contributing to polygenic disorders through collective interaction (figure 3). ALS-associated genes are typically rare variants that can display variable penetrance. For example, a rare variant in a gene, such as *SOD1* A5V, can act as a highly penetrant allele, leading to a clear dominant pattern of inheritance in which the variant has a strong effect size in causing ALS. Other rare variants in the same gene, such as *SOD1* I114T, probably act as cofactors requiring other biological steps, and therefore manifest with variable penetrance in which some gene carriers never develop the disease, which may skip generations. Penetrance can be influenced by the presence of other genotypes or their variable expression, which may depend on epigenetic, environmental and stochastic events. Penetrance may also vary for the same genotype between pedigrees, and estimates based on familial cases seen in clinics can thus be biased. ‘Sporadic’ ALS is still likely to have a significant genetic contribution, but from multiple rare and common variants of individually small effect sizes in a combination which is not passed on intact, and therefore not associated with a family history.

Although there are no gold standard measurement techniques, crude penetrance estimates for a specific genotype can be derived from a disease population by dividing the observed number of individuals with the disease (penetrant) by the total number of obligate carriers (non-penetrant and penetrant).^34^ In UK MND cohorts, this has revealed a high penetrance for some *SOD1* (e.g., A5V) and *FUS* variants,^28^ but significantly incomplete penetrance for the *C9ORF72* hexanucleotide repeat expansion.^33^ However, penetrance is clearly age-dependent, with near complete expression (99.5%) of the phenotype reported by 83 years for carriers of the latter, although from studies limited to single pedigrees with inherent bias. Penetrance has also been reported to be higher within an affected family of *C9ORF72* hexanucleotide repeat expansion carriers, independently of the genotype-specific rate.^28^ Penetrance estimates must therefore be appropriately tempered by the family-specific context when interpreting a positive result in the clinical setting.

Several other factors may have associations with penetrance. When interrogating the history of earlier generations (particularly pre-1930s), carriers may have had an equal chance of dying from another disease due to shorter life expectancies (Gompertzian inter-disease competition phenomenon).^28^ Other factors include site of symptom onset and sex, with higher penetrance noted for limb-onset versus bulbar-onset cases and in males versus females, although the cause of such variation is unclear, with epigenetic and lifestyle factors likely.^28 33^ Importantly, separation into familial or sporadic groups has not been shown to influence penetrance in *C9ORF72*,^33^ corroborating the arbitrary nature of such division.

#### Variants of uncertain significance

The human genome contains approximately 3 billion base pairs. Between any two individuals, the amount of variation is only 0.1%. The most common sources of variation are single base pair differences (termed single nucleotide polymorphisms), but other less common differences include copy number variation, insertions, deletions, duplications and rearrangements. An individual’s DNA carries thousands of variants, which relies on comparison with large population databases. Variants can be classified as:

1. Pathogenic.
2. Likely pathogenic.
3. Variant of uncertain significance.
4. Likely benign.
5. Benign.

The categorisation of a genetic variant is ultimately a probability estimate according to consensus criteria. Ideally, this includes evidence of significance in a case–control study, though this is not always available for ALS variants. It may also include population database frequencies and evidence from prior clinical reports. This is particularly relevant where there is clear co-segregation of the variant and disease in multiple family trees, ideally of varying ethnicity.^35^ While a specific reproducible molecular signature derived from a laboratory test or animal model can also support pathogenicity, this rarely provides sufficient evidence in most autosomal dominant ALS mutations, where a definitive disease mechanism is not available. If a variant still does not fulfil either the benign or pathogenic classification, or if the evidence for either is conflicting, it defaults to that of uncertain significance. Within *SOD1*, there are currently more than 200 variants listed (www.alsod.ac.uk). Not all of these carry the same level of statistical confidence that they are pathogenic, with a wide variation in penetrance and phenotype.^36 37^ Several international population databases aggregate known variant information and are publicly available (eg, ClinVar, https://www.ncbi.nlm.nih.gov/clinvar).

With the increasing availability of whole-genome sequencing, including its speculative use outside traditional healthcare settings, a variant of uncertain significance will become a common challenge for clinicians of all specialities.^35^ Indeed, the availability of a ‘virtual panel’ to test specific genes linked to ALS has enabled targeted bioinformatics analysis of raw sequence data including the uninterpretable data files, increasing the likelihood of generating incidental and unsolicited findings. This multigene panel testing suggests a high rate of variants of uncertain significance (up to 30%) in patients with a clear family history,^38 39^ and undoubtedly this will arise more frequently with the more routine application of screening to all those diagnosed with ALS. Additionally, even among experienced geneticists, significant variation in applying consensus criteria exists, with a small proportion of differences potentially affecting medical management.^40^ Counselling will require adequate training and time to explain complex biological as well as statistical concepts, acknowledging the limits of certainty with confidence while minimising psychological harm to both the affected individual and their asymptomatic relatives. It is likely that this will require close collaboration with genetics consultants for most neurologists.

#### Insurance and discrimination considerations

The UK Government and Association of British Insurers published a voluntary ‘Code on Genetic Testing and Insurance’ in 2018 (https://assets.publishing.service.gov.uk/government/uploads/system/uploads/attachment_data/file/751230/code-on-genetic-testing-and-insurance.pdf) to apply to life insurance, critical illness insurance and income protection insurance. The results of diagnostic genetic tests form part of the relevant medical information in such applications in the same way as for blood tests and scans, and the wider family history of medical conditions must be disclosed. It is the use of predictive genetic testing that the code seeks to define. Such tests are only considered in an application for the largest policies and only for conditions designated as ‘highly predictive of a relevant risk’. At present, only Huntington’s disease for life insurance totalling more than £500 000 per individual is listed. Insurers agree not to require or pressure applicants to undertake any type of genetic test. Predictive tests voluntarily purchased or taken on the recommendation of a clinician are covered by the code, but genetic tests on an individual in the context of scientific research do not need to be disclosed.

The USA’s 2008 Genetic Information Non-Discrimination Act bars the use of genetic information in both health insurance and employment settings based solely on genetic predisposition in someone without symptoms, with a few important exemptions (such as the Military). The Council of Europe’s 2016 recommendation CM/Rec(2016)8 similarly sets out limits in processing personal health-related data for insurance purposes, including data resulting from genetic tests (https://search.coe.int/cm/Pages/result_details.aspx?ObjectId=09000016806b2c5f). The Genetic Alliance UK website provides additional guidance in relation to genetic testing and insurance cover (http://www.geneticalliance.org.uk/information/living-with-a-genetic-condition/insurance-and-genetic-conditions/).

### Testing framework

Historically, clinical genetics consultants have been the gatekeeper to both diagnostic and predictive testing for many disorders, as well as the source of counselling in the UK. We suggest that their expertise remains the gold standard in testing asymptomatic individuals and around issues of family planning (see later). However, the international momentum towards more widespread use of genetic screening within secondary care means that their capacity is likely to be rapidly outstripped by need, making it incumbent on all neurologists to become familiar with the broad issues. It should be made very clear to patients considering genetic testing that it is not being offered on a research basis.

In practical terms, at least one-third of those diagnosed with ALS who have a clear dominantly inherited ALS pedigree will currently have no identifiable genetic cause, though this does not reduce the risk of onward heredity. It is easy to underestimate the sense of disappointment this lack of molecular certainty entails, and individuals must be counselled about this before testing. Within the much larger apparently sporadic case population (~90%), a pathological variant will be identified in at least 10% of cases, with some studies proposing more than a fifth with clinically actionable findings.^41 42^ Even using the conservative estimate, 10% of the apparently sporadic cases of ALS is a greater number than 70% of the cases who report a family history. In anticipation of increasing potential for genetic trial participation, and without any hesitation should a therapeutic agent emerge, we suggest that offering genetic screening should now be a routine part of the management for all newly diagnosed cases of ALS regardless of the presence, or not, of a positive family history. Furthermore, we suggest that the rigid use of patient age (e.g. less than 50 years old) as a criterion for genetic testing decisions is scientifically flawed as well as potentially disenfranchising.

As the most common in UK clinics, the *C9ORF72* hexanucleotide repeat expansion is the first to be tested in someone diagnosed with ALS (with or without FTD). A dedicated assay is required to detect repeats of this nature and size, which more recent automated sequencing methods may miss. A negative result will typically trigger the screening of a larger panel of ALS-associated genes (online supplemental table 1). It is not currently usual practice to look for additional genes if the *C9ORF72* hexanucleotide repeat expansion is detected. Clinically available testing options have now widened in some countries to include whole-exome sequencing with the direction of travel towards whole-genome approaches, which will become more routine in the future as methods mature for generating polygenic risk scores across many genetic variants.

10.1136/practneurol-2021-002989.supp1 Supplementary data

We now discuss the value of genetic testing in the context of different ALS-related scenarios.

#### In the diagnosis of ALS (of no value)

The diagnosis of ALS fundamentally remains a clinical one. For a complex disorder in which the genetic contribution is both limited and not fully characterised, there is no role for speculative genetic testing in the diagnostic pathway, with neither rule-in nor rule-out value. This applies equally to the concerned relative of an established patient with MND, who should be assessed on clinical grounds regardless of whether or not a causative gene has been established.

#### For the individual diagnosed with ALS (the value depends on the context)

After a diagnosis of ALS, the discussion might focus on the value of a molecular diagnosis in potentially facilitating entry into a therapeutic trial (or in benefiting quickly from an emerging gene-based therapy). However, many people place value on simply trying to understand as much as they can about the cause of their condition or in having all data available for the potential benefit of future generations of their family, regardless of the opportunity for trial participation.^43^ Setting the correct expectations in either case is important when discussing genetic testing, and there are several points of consideration. For an individual with ALS with an affected first-degree relative, the pretest probability of a positive genetic test is currently ~70%. For those with extensive negative family history knowledge (i.e., apparently sporadic patients), this is much nearer ~10%. For an individual lacking clear knowledge of their wider family, it is somewhere in between. For patients who may never have supposed the possibility of heredity, this news may present a confronting concept and an uncontemplated burden of ‘genetic responsibility’. Further empirical data on these patients’ perspectives are warranted, although most attitudes are reported as favourable and increasingly buffered by a possibility of inclusion in gene therapy trials.^43^ The discussion should be tempered by the fact that there is currently no highly effective therapy, genetic or otherwise, for ALS, and no guarantee of entry into a trial. Information about uncertainties on penetrance (see above) and in predicting prognosis (see later) should also be included, and patients may question the implications for family members (see below). Finally, an individual must also have the mental capacity to make an informed decision to have genetic testing.

Any wider value of screening the affected patient for their relatives, such as for guiding their family planning, must be weighed carefully with the significant potential for asymptomatic relatives to feel ambushed by the news that they are at an increased risk of being a carrier of the same pathological variant. For those who have an extensive family history of ALS (±FTD), relatives will often already be aware of an inherited component to the condition. An important consideration in such cases is that there is currently still a 30% chance that testing will fail to identify a precise genetic cause, which can be an equally unsettling outcome by lacking a sense of closure. As such, affected individuals undergoing genetic testing are advised to wait for the results and discuss their implications with the clinician before revealing to other family members that they have even requested testing. This can present a practical difficulty in the clinic if they are accompanied by others, who may have a strong personally driven interest in encouraging or dissuading the affected individual from testing.

The option of just DNA storage for potential future testing is a routine service offered by most clinical genetics departments. It is a useful option where an individual with ALS remains unsure after counselling or specifically wants any testing to only occur after their death if a family member wishes to access the sample.

#### In predicting prognosis for a patient with MND (of very limited value)

MND encompasses a range of clinical phenotypes with variable lower or upper motor-neurone-dominance, and this cannot be predicted by genotype. For example, primary lateral sclerosis is a very rare and distinct ‘pure’ upper motor neurone degenerative disorder^44^ that can be challenging to distinguish from upper motor neurone-predominant forms of ALS or hereditary spastic paraparesis, but monogenetically determined cases have not been convincingly described.

Particular genotypes have revealed more consistent rates of disease progression, as exampled by the *SOD1* A5V (typically fast) versus D91A (typically slow) variants.^37^ Although severe cognitive impairment in ALS is unusual and often apparent at or soon after motor presentation, it is more consistently associated with the *C9ORF72* hexanucleotide repeat expansion and conversely very rare in *SOD1* gene variants.^45^ In general, however, genotype does not consistently allow prediction of the clinical disease and rarely surpasses the value of prognostication via clinical means, such as the interval from symptom onset to diagnosis (which is proportional to survival) and the age at symptom onset (inversely proportional to survival).^46^ We therefore advise against genetic testing solely for prognostication purposes.

An emerging potential for a more routine use of DNA sampling at diagnosis in ALS is in pre-identifying ‘therapeutic responders’ based on genetic subclass. An example came from the post hoc analysis of lithium treatment in ALS, which revealed a survival benefit for a subgroup with the *UNC13A C/C* genotype, despite an overall negative result from the trial,^47^ though this has not resulted in different treatment by genotype in practice.

#### For asymptomatic relatives (refer to clinical genetics)

In the absence of preventative therapy and with the added uncertainties of penetrance, testing the asymptomatic relatives of those diagnosed with ALS has great potential to do harm if not carefully counselled, as is well understood from the Huntington’s disease experience. On current knowledge, anyone can develop ALS, with an overall lifetime risk of approximately 1 in 400. The lifetime risk of ALS for first-degree relatives of an apparently sporadic case of ALS is only slightly increased overall (<3%) and is even lower for second-degree relatives, with no significant increase for more distant relations.^48^

In the case of a clearly dominant pedigree of ALS (which might include cases with pure FTD), the asymptomatic first-degree relative has a 50% chance of being a carrier and, if so, a significantly higher chance of developing ALS (or FTD in the case of the *C9ORF72* hexanucleotide repeat expansion) in a natural lifespan, with the caveat that penetrance is not 100% (see earlier). The common motivation for asymptomatic individuals to seek predictive testing is to try to reduce anxiety or uncertainty about the future and possibly plan their lifestyle accordingly, and predictive testing requests are steadily increasing. Only for the scenario in which the genotype of the affected relative is known may the first-degree relative gain certainty about their carrier status and, if negative, that their lifetime risk of ALS is then the same as that of the general population. This level of chance is undoubtedly a powerful temptation that is hard to weigh equally against the 50% chance of a positive carrier result and its frequently devastating consequences on mood (despite efforts to convey the significant uncertainty around penetrance). In cases where the precise genotype in a positive family history is not known, the value of panel screening for the asymptomatic individual is even more limited, given the 30% failure to identify a gene even in dominant pedigrees.

It is our practice to consistently involve specialised genetic consultants in any request for asymptomatic testing, which also incorporates a ‘cooling off’ period between their counselling and any sample being taken. This particularly applies to the highly specialised area of pregnancy and family planning once the causal mutation is known, with options including prenatal testing (and subsequent termination if the variant is identified) and pre-implantation genetic testing for monogenic disorders (PGT-M) through embryo selection and in vitro fertilisation. In the UK, the latter is overseen and regulated by the Human Fertilization and Embryology Authority (https://www.hfea.gov.uk/) and in theory, may be undertaken without the need to disclose carrier status to the at-risk parent (see cases later).

#### For presymptomatic research participation (blinded)

Any aspiration for the prevention of ALS will depend on understanding the presymptomatic changes. The study of asymptomatic carriers of pathogenic variants has already revealed important potential biomarkers.^49 50^ In our experience, close relatives of affected familial ALS cases are highly motivated to take part in research, but the majority do not wish to know their own genetic status. We suggest that it is essential for the research study team (whose members often undertake clinical care of the index case) to also remain blind to the participant relative’s carrier status, to avoid accidental disclosure or the perception by the participant of having undisclosed awareness. This requires a third party ‘data guardian’ who can allocate anonymised data to carrier and non-carrier groups for analysis.^51^

Some challenging scenarios are considered with practice points (see box 1).

Box 1 **Some challenging scenarios in the clinic**

**Scenario 1.** The two children of a patient with ALS without a wider family history accompany their parent (mother) to clinic. After routine discussion about the causes of ALS, the patient expresses a wish to have all known genetic causes excluded. One of the children becomes angry, saying not to do this as he does not want to find out that he is at an increased risk of getting the disease.

- **Comment:** Professional ethics codes support the clinician’s primary obligation towards the patient’s autonomy, but this presents a practical difficulty if not aligned with a close family relative for whom a positive result may have a personal impact.
- **Practice points:** Testing motivations should be explored with the patient within their family-specific context. The ‘right not to know’ is rooted in personal preferences and supported by most national legislation, but this may change in the setting of an effective treatment option. Importantly, the choice to disclose a carrier status to family members is currently left at the patient’s discretion, with no clinical ‘Duty to Warn’ in the absence of therapy. Expectations of testing need to be clarified, including the chance of getting no result (even in the setting of a dominant history), the caveats of variant interpretation and the impossibility of excluding all possible genetically mediated influences. Tailored discussion can occur separately for parent and child and over several encounters. Clinical genetics counsellors are likely to be particularly useful in exploring complex family dynamics and helping to resolve differences.

**Scenario 2.** A 16-week pregnant woman with ALS without a family history requests rapid genetic testing to guide termination if an ALS gene variant is found.

- **Comment:** This forms a key reason for predictive testing requests in ALS. Motivations for termination are primarily due to concern that the child may get the disease and are particularly influenced by a personal experience with the disease (e.g., as a carer, or through the loss of a loved one).
- **Practice points:** This is best supported through referral to specialised genetic services. Prenatal testing cannot be undertaken in this case without a priori knowledge of a pathogenic risk variant within the family. If an X-linked pathogenic variant is identified in the patient (whereby males are typically affected, eg, *UBQLN2*), fetal sex determination may help guide decisions around termination. The limitations of testing the patient, however, include the low pretest probability of finding a pathogenic result (assuming the family history is extensively negative) combined with the higher likelihood of finding an uninterpretable variant. That the absence of a result does not exclude the genetic basis of disease must also be communicated, all of which complicates translation to fetal carrier risk.

**Scenario 3.** A 30-year-old patient with ALS but without a family history requests genetic testing and is found to have a C9ORF72 hexanucleotide repeat expansion. At a subsequent appointment, the patient is accompanied by both parents, who are asymptomatic.

- **Comment:** One biological parent must be an obligate carrier in this case, as large hexanucleotide repeat expansions are not thought to occur de novo. This highlights the real-world complexities of disease manifestation in gene carriers, as well as the limitations of family history taking and ‘skipped generations’.
- **Practice points:** Consideration must be given to non-paternity, which might be unknown to both the parent and affected patient. An explanation of the risk to an obligate carrier parent of developing the clinical disease must be given in the context of variable penetrance. This suggests that this risk is higher in later life but is not certain.

**Scenario 4.** A patient with ALS without a family history and with a lower motor neurone-predominant phenotype arranges private whole-genome sequencing, which reports a variant of uncertain significance in *ABCD1*. The patient is concerned that they have been misdiagnosed.

- **Comment:** Variants in the *ABCD1* gene have been linked to adrenomyeloneuropathy (AMN), a slowly progressive pure upper motor neurone degenerative disorder.
- **Practice points:** In this case, the patient’s clinical syndrome was not in keeping with adrenomyeloneuropathy and the *ABCD1* gene is not known to independently influence either the onset or prognosis of ALS. Multiple pathogenic variants in more than one gene associated with motor system degenerative disorders are increasingly recognised, but this finding still seems overwhelmingly likely to be incidental. Discussion with the national reference laboratory was also helpful in this case and revealed that the apparent variant in *ABCD1* was a well-recognised incidental finding. The patient was not seeking advice about family planning, and this finding is of no practical relevance. This highlights the future challenges of more routine whole-genome sequencing in healthcare.

**Scenario 5.** A patient with ALS without a family history and with a typical progression rate has read about gene-targeting trials in SOD1-mediated ALS. They request genetic testing so that they might be eligible to enrol. It reveals that they are heterozygous for a ‘likely pathogenic’ variant associated with a characteristically very slowly progressive, typically homozygous recessive form of ALS.

- **Practice points:** A major drive by patients for more routine genetic testing of all new diagnoses of ALS is to enable enrolment in gene-targeting therapy trials. It is extremely important to control expectations around the testing positivity rate, the eligibility criteria for any given study and the uncertainty of drug effects at present. In the setting of novel variants of uncertain significance where the clinical phenotype is not helpful, most clinical genetics laboratories offer family testing for variant reclassification (although this is not a guaranteed outcome), but eligibility criteria differ across testing facilities. The need to identify and test other family members as part of this process may present practical limitations.
- **Comment:** There are more than 200 variants listed in *SOD1,* linked to variable rates of progression. The pathogenicity of many variants has not yet been established. Antisense oligonucleotide trials to date have limited their enrolment criteria to dominant variants that are firmly associated with ALS and of typical progression rate, in order to maximise the power to show a treatment effect.

**Scenario 6**. An at-risk (but untested) relative wants blinded pre-implantation embryo selection.

- **Comment:** If the causal genetic variant within the family is known, pre-implantation genetic testing can be done for a single-gene defect (PGT-M) and subsequent embryo selection can be undertaken without the need to disclose carrier status.
- **Practice points:** This highly specialised issue requires guidance through clinical genetic services. In the UK, requests for pre-implantation genetic testing of the specifically known variant (e.g., SOD1 A5V) must be formally approved under the Human Fertilisation and Embryology Act 2008 (HFEA, https://www.hfea.gov.uk/pgt-m-conditions/). Testing is carried out via licenced genetic clinics and unaffected embryos can be selected for in vitro fertilisation (IVF). At-risk individuals can avoid knowing their carrier status through grandparental exclusion studies, which is now preferred to result non-disclosure. The issue of conferring ‘genetic responsibility’ to the child not at risk of the familial condition has been raised as a possible concern, and this discussion must be incorporated into pretesting genetic counselling.

## Concluding remarks

The absence of a proven gene-targeting therapy is now the only factor preventing the mandatory offering of routine genetic testing to all those diagnosed with ALS, regardless of family history. However, there are compelling reasons why this approach is already of value, and the direction of travel towards wider involvement of genomics in healthcare seems certain. As for any medical test, the limitations and potential for harm from genetic screening must be fully understood by the requesting clinician. They must also be willing and able to counsel the individual accordingly and offer prompt referral to specialised genetic services as required.

**Key points**

- There is no role for genetic testing in the diagnostic pathway of amyotrophic lateral sclerosis (ALS).
- At least 10% of those with apparently sporadic ALS have a monogenetic variant of likely significance, and the distinction of familial versus sporadic is misleading.
- An intronic hexanucleotide repeat expansion in *C9ORF72* is the most common genetic cause of ALS in European populations, but is much rarer in Asian populations.
- A dominant family history including cases suspicious for frontotemporal dementia makes an underlying *C9ORF72* hexanucleotide repeat expansion likely, and the pedigree may include neuropsychiatric conditions.
- We suggest offering genetic testing to all newly diagnosed ALS cases (i.e., regardless of family history or patient age) after an appropriate level of discussion around the value and potential drawbacks, or the option of providing a DNA sample for storage with the potential to request testing in the future.
- There is currently no recommendation for genetic testing of asymptomatic relatives of patients with ALS, who should be referred to specialised genetics services for both this and wider issues around family planning.
