# Supplementary material for: Genetic testing in motor neurone disease
Source: Pract Neurol. 2022 Jan 13;22(2):107–16. doi: 10.1136/practneurol-2021-002989 (PMC8938673; doi:10.1136/practneurol-2021-002989)
Supplement: Supplementary data [file practneurol-2021-002989supp001.pdf]

Supplementary Table 1: UK gene testing for ALS/MND

	Gene	OMIM*	Inheritance	Chromosome	Penetrance	Variant details	ALS clinical traits	Other associated disorders
	C9ORF72 (chromosome 9 open reading frame 72)	614260	AD	*9p21.2*	Incomplete	STR:G4C2<br>N: ≤30<br>Abn[#]: >100	Mainly of European (North>South) ancestry; Younger adult onset, rapid progression, extra-motor symptoms common (cognitive, psychiatric)→ALS-FTD	Dementias: FTD (bv, PPA), AD; Parkinsonism (typically akinetic rigidity without tremor); Schizophrenia
ALS/MND sequencing slice:								
**1**	*ALS2 (KIAA1563, ALSIN)*	606352	AR	*2q33.1*	Complete	Never reported in adult patients	Mainly of Arabic ancestry; Juvenile onset, long ddx, often no bulbar/respiratory sx	jPLS; jHSP
**2**	*ANG* (angiogenin)	105850	AD	*14q11.2*	Complete			Parkinsonism; FTD
**3**	*ANXA11* (annexin XI)	602572	AD	*10q22.3*	Incomplete	4 mutations **	Classic ALS phenotype	
**4**	*CCNF* (cyclin F)	600227	AD	*16p13.3*	Incomplete		ALS, ALS-FTD	FTD
**5**	*CHCHD10* (coiled-coil-helix-coiled-coil-helix domain-containing protein 10)	615903	AD	*22q11.23*	Complete		FTD-ALS	FTD; Mitochondrial myopathy, SMA (Jokela type)
**6**	*CHMP2B* (charged multivesicular body protein 2B)	609512	AD	*3p11.2*	Complete		LMN-predominant ALS phenotypes; FTD-ALS	FTD
**7**	*FIG4* (FIG4 phosphoinositide 5-phosphatase)	609390	AD	*6q21*	Complete			*AR:* CMT-4J
**8**	*FUS/TLS* (fused in sarcoma/translated in liposarcoma)	137070	AR<br>AD	*16q11.2*	Complete		Juvenile onset;<br>Heterogenous phenotype	Essential tremor type 4
**9**	*HNRNPA1* (heterogeneous nuclear ribonucleoprotein A1)	164017	AD	*12q13*	Complete			IBMPFD
**10**	*OPTN* (optineurin)	602432	AD<br>AR	*10p13*	Complete		Mainly in Japan	*AD:* Glaucoma 1,open angle, E
**11**	*PFN1* (profilin-1)	176610	AD	*17p13.2*	Incomplete			
**12**	*SETX* (senataxin)	608465	AD	*9q34.13*	Complete		Juvenile onset; very slow progression (normal lifespan), no bulbar/ respiratory sx	dHMN;<br>*AR:* SCAR1; AOA2
**13**	*SOD1* (superoxide dismutase 1)	147450	AR<br>AD	*21q22.11*	Complete	>200 mutations**	Younger onset; longer ddx, LL-onset; rare to have cognitive sx	*AR:* Spastic tetraplegia and axial hypotonia
**14**	*SPG11* (spatacsin)	610844	AR	*15q21.1*	Complete		Juvenile onset (<25yrs); slowly progressive, prolonged survival (>3 decades)	CMT-2X; HSP+-TCC
**15**	*SQSTM1* (sequestosome 1)	601530	AD	*5q35.3*	Complete		ALS-FTD	FTD; Distal myopathy, Paget disease of the bone

**16**	*TARDBP* (TAR DNA-binding protein 43)	605078	AD	*1p36.22*	Incomplete	Polygenic	Mainly of Sardinian ancestry; ALS-FTD	FTD
**17**	*TBK1* (TANK-binding kinase 1)	604834	AD	*12q14.2*	Incomplete	Not frequent	ALS-FTD	FTD
**18**	*UBQLN2* (Ubiquilin-2)	300264	XL	*Xp11.21*	Complete		Typically in males, if in females may be less severe, later onset; ALS-FTD	
**19**	*VAPB* (vesicle-associated membrane protein B angiogenin)	605704	AD	*20q13.32*	Complete	P56S mutation (mainly)	Longer ddx	SMA (late onset, Finkel type)
**20**	*VCP* (valosin-containing protein)	601023	AD	*9p13.3*	Complete	Rare (<2%)	ALS-FTD	CMT-2Y (axonal); HSP; IBMPFD

In the UK, the genetic sequencing slice for ALS/MND is available as part of the Adult-Onset Neurodegeneration panel (R58.1, NHS Genomic Medicine Service v2.178) if testing for *C9ORF72* is negative. This contains 20 genes with a "high level of diagnostic evidence" for ALS and comprises whole genome sequencing (WGS phase 2, 2021), short tandem repeats (STRs), and curated regions (copy number variations). ALS/MND gene testing panels differ across countries according to clinical laboratory and regional practice (e.g., the College of American Pathologists accredit a 35-panel test across North and South America, Europe, Australia and the Middle East; while Centogene (Germany) offer a 22-panel test). Row colours separate mode of inheritance: AD, autosomal dominant (blue); AR, autosomal recessive (orange); XL, X-linked (green); Abn, abnormal range; ALS, amyotrophic lateral sclerosis; ALS-FTD, amyotrophic lateral sclerosis with frontotemporal dementia; AOA2, ataxia with oculomotor apraxia type 2; bv, behavioural variant FTD; CMT, Charcot-Marie-Tooth disease; ddx, disease duration; dHMN, distal hereditary motor neuropathy; FTD, frontotemporal dementia; HSP, hereditary spastic paraplegia; HSP+, complicated hereditary spastic paraplegia (HSP-plus); IBM, inclusion body myositis; IBMPFD, inclusion body myopathy with Paget Disease and Frontotemporal Dementia; j, juvenile; LL, lower-limb; N, normal range; PPA, primary progressive aphasia (language variant FTD); PLS, primary lateral sclerosis; SCAR1, autosomal recessive spinocerebellar ataxia with axonal neuropathy; SMA, spinal muscular atrophy; sx, symptom(s); TCC, thin corpus callosum. *Gene/locus MIM number; #, no validated or exact cut-off length for pathogenicity, but usually >100; **pathogenicity not proven for all mutations (SOD1: www.alsod.ac.uk).
